# Consumptive hypothyroidism complicating infantile hepatic hemangioma successfully treated with propranolol: a case report and literature review

**DOI:** 10.1186/s13052-025-02020-9

**Published:** 2025-06-23

**Authors:** Irene Bettini, Giulia Poletti, Alessandro Rocca, Valeria Di Natale, Monia Gennari, Marcello Lanari, Andrea Pession, Alessandra Cassio

**Affiliations:** 1 Specialty School of Pediatrics, Alma Mater Studiorum, University of Bologna, Bologna, Italy; 2 Pediatric Emergency Unit, IRCCS, Azienda Ospedaliero-Universitaria di Bologna, Bologna, Italy; 3 Pediatric Unit, IRCCS, Azienda Ospedaliero-Universitaria di Bologna, Via Massarenti 11, Bologna, 40138 Italy

**Keywords:** Case report, Consumptive hypothyroidism, Infantile hepatic hemangioma, Propranolol, Thyroid hormones

## Abstract

**Background:**

Consumptive hypothyroidism (CH) is a rare paraneoplastic syndrome, caused by the overexpression in vascular tumors of type 3 deiodinase (D3), converting thyroid hormones into inactive metabolites.

**Case presentation:**

We report the case of a 2-months-old male infant with diffuse infantile hepatic hemangioma (IHH). Thyroid function screening detected a CH. The patient was promptly treated with propranolol: after 2 weeks, a marked reduction in tumour size was observed and thyroid function was restored. No hormone replacement therapy was required. We then performed a literature review on PubMed/Medline: based on the title and abstract, we extracted 64 cases of CH secondary to IHH published between 2000 and 2023. 59.37% patients received propranolol, either alone (52.63%) or in combination with other treatments. 18.75% patients required surgical treatment or liver transplant. LT4 was administered in 92.85% of the patients. Patients who received propranolol required a lower dosage of LT4 than patients who received other treatments.

**Conclusions:**

Thyroid function should be evaluated in all children with IHH to rule out CH. Early recognition of IHH and CH and prompt therapy with propranolol can effectively treat IHH and the subsequent hypothyroidism, sometimes even without the need of hormone replacement therapy.

## Background

Infantile hemangioma (IH) is a vascular neoplasm characterized by abnormal proliferation of endothelial cells and aberrant blood vessels architecture [1]. IH is the most common benign tumors of childhood, occurring in about 5% of children [2]. The neoplasm probably develops from placental stem cells embolized during early fetal life to tissues where placenta-like growth is possible, such as the skin and the liver. IH usually appears before 4 weeks of age and then shows a rapid growth up to 12 months of age, followed by a slow involution phase. A minority of cutaneous IHs are potentially problematic, due of their size or position [3–6]. Infantile hepatic hemangioma (IHH) is the most common visceral IH, and typically occurs in patients with multiple cutaneous IH [2, 7]. Diffuse IHH has a significant morbidity and mortality due to complications such as hepatomegaly and abdominal compartment syndrome [1, 6, 8–10]. 

A recently identified paraneoplastic syndrome, defined consumptive hypothyroidism (CH), can complicate diffuse IHH [11]. CH results from the over-expression in the endothelium of vascular neoplasm of iodothyronine deiodinase type 3 (D3), which converts thyroid hormones into inactive metabolites: T4 into reverse T3 (rT3) and T3 into T2. D3 is physiologically expressed in fetal tissues and in the placenta, having the role of protecting the fetus from excesses of thyroid hormones, while during extrauterine life its expression is limited. CH due to D3 overexpression is characterized by increased TSH and rT3 levels, with normal or low fT4 and fT3 [12]. 

After the serendipitous observation of the usefulness of propranolol in reducing the size of cutaneous IH [13], propranolol has become the treatment of choice for cutaneous IH [2, 14] and has been increasingly used for the treatment of IHH [10, 15–17]. Propranolol acts by inducing vasoconstriction and inhibition of angiogenesis [1]. It is a safe and effective therapy, with rare side effects [18, 19]. 

CH is treated with substitutive therapy: the required replacement dose of levothyroxine (LT4) is usually much higher than the replacement dose in congenital hypothyroidism, due to massive peripheral conversion to inactive forms. In severe cases, therapy with liothyronine can be associated [12]. 

We report a case of IHH complicated by CH, completely recovered after treatment with oral propranolol without the need of thyroid hormone replacement. We also perform a literature review on CH-complicated IHH to evaluate existing cases, proposed treatments, as well as follow up and outcomes. We searched PubMed and Medline databases for studies published between 2000 and 2023 about IHH and CH. The complete search items were: (children OR child OR infant OR infantile OR pediatric OR paediatric OR newborn) AND (hepatic hemangioma* OR hepatic haemangiom* OR hemangioendotheliom* OR haemangioendotheliom* OR haemangiom* OR hemangiom*) AND (consumptive hypothyroidism OR hypothyroidism). References within the included articles were then reviewed and additional case reports were identified. Only articles in English were accepted. Two reviewers independently selected articles by title, abstract and full paper review. We included 46 case reports or case series, for a total of 64 patients with IHH complicated by CH. Demographic, clinical, laboratory and radiological, treatment and outcome data were collected for all articles matching the criteria on an electronic spreadsheet. Data were extracted by one reviewer and checked by a second.

## Case presentation and literature review

A 2-month-old male infant was referred to our Centre in April 2020 because of poor feeding and failure to thrive. The baby was born at 36 weeks of gestational age after a normal pregnancy, one of two twins conceived through egg donation. His birth weight was 2120 g (< 3rd percentile). Prenatal ultrasounds were normal and his twin was healthy. Neonatal screening for TSH level was normal (TSH 2.71 microU/ml, n.v. < 10). From two weeks of age the patient was irritable after meals, with frequent regurgitations and emission of liquid and malodorous stools 6–7 times/day.

**Fig. 1:**
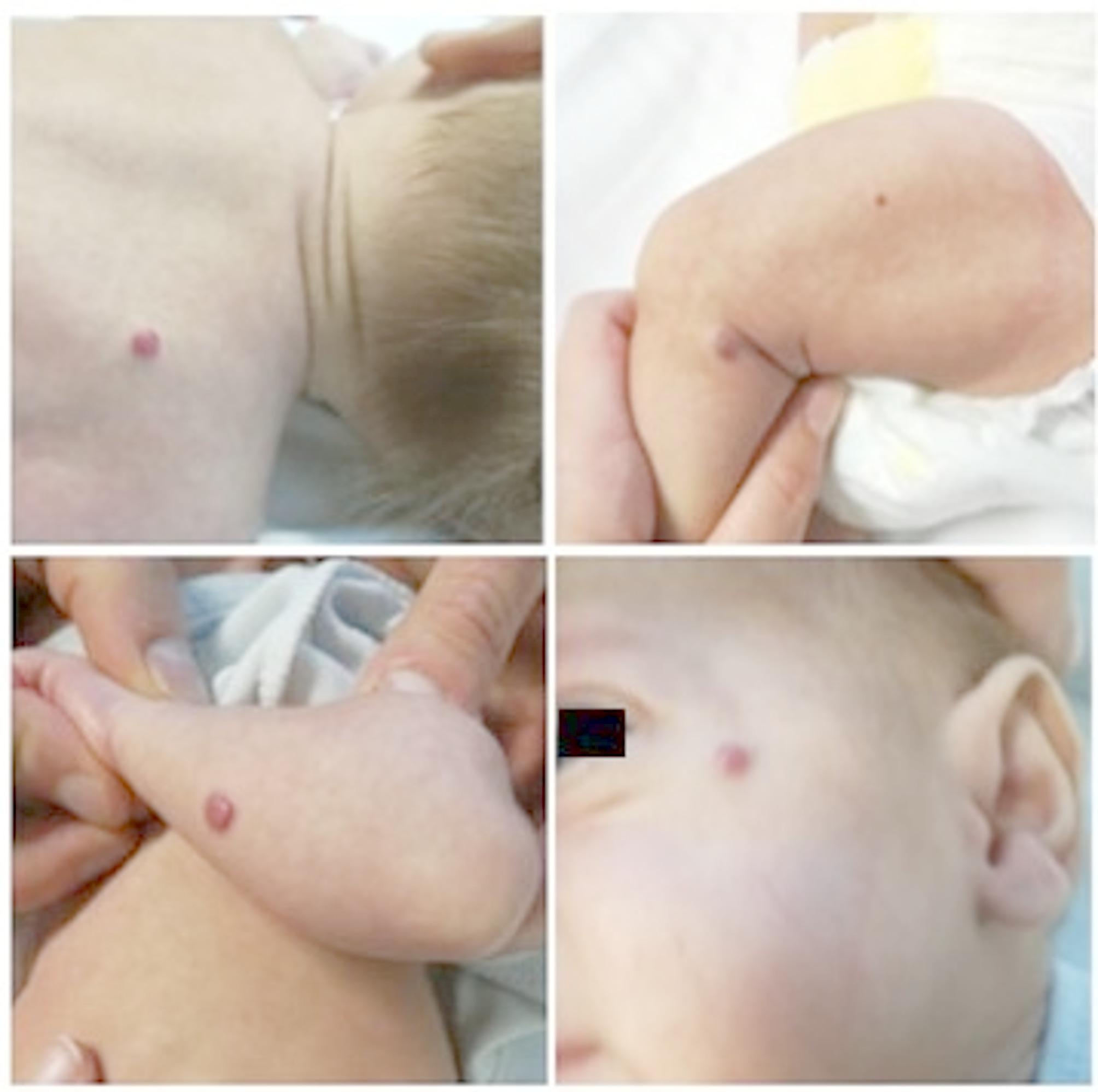
Multiple cutaneous hemangiomas detected at first examination of the patient

**Fig. 2:**
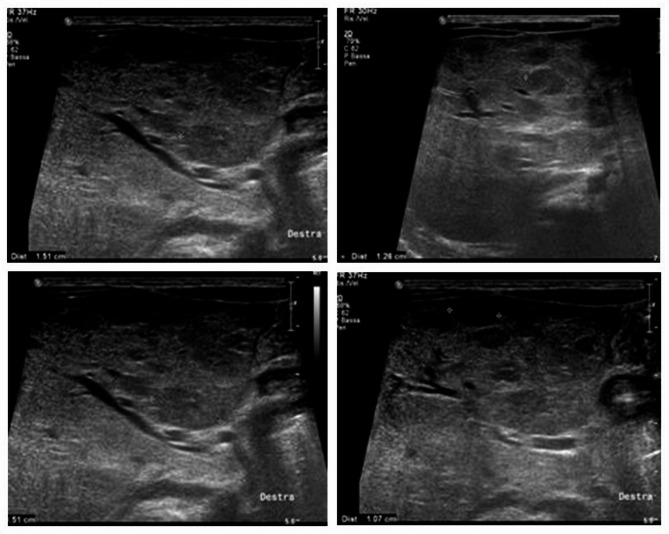
Abdominal ultrasonography at diagnosis showing slight hepatomegaly with well-delineated uncountable hypo-isoechoic lesions with a mean diameter of 15 mm

**Fig. 3:**
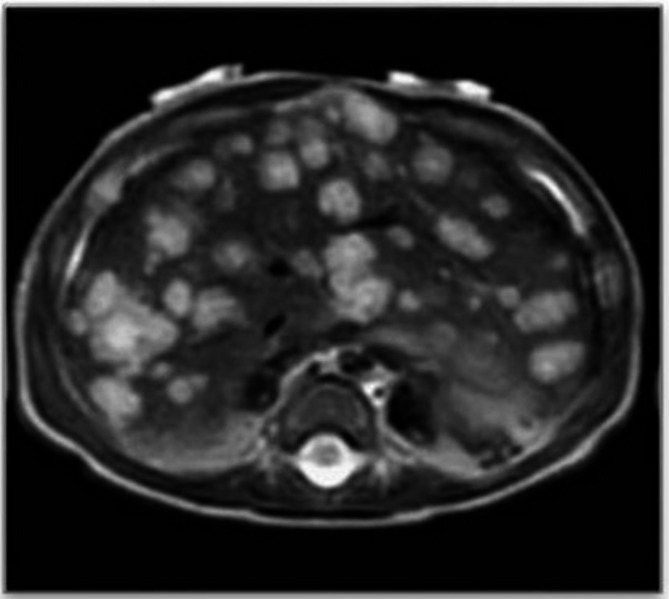
RMI at diagnosis showing hepatomegaly, with diffusely distributed nodules with maximum diameter of 20 mm, hyperintense in T2-weighted images, with nearly total replacement of the hepatic parenchyma, indicative of diffuse IHH

On first examination, the child was pale and 10 small cutaneous IHs (diameter < 5 mm) were observed on the face, chest wall, back and limbs; the parents reported that they had grown in size since birth. (Fig. 1) The remainder of the physical examination was normal. His weight was 3390 g (< 3rd percentile). The blood test revealed normocytic anemia, a slight electrolyte imbalance with a Na level of 134 mmol/L, normal renal and hepatic function, and elevated alfa-fetoprotein (13923 ng/ml, normal value < 10). Cardiac function was normal. We suspected IHH, so abdominal ultrasound and MRI were performed and showed marked hepatomegaly with diffuse nodules with a maximum diameter of 20 mm with nearly total replacement of the hepatic parenchyma, indicative of diffuse IHH. (Figures 2 and 3) To rule out a CH, thyroid function was tested: TSH, fT4, fT3 and rT3 values at diagnosis and during follow-up are shown in Table 1. A normal in situ gland showed at ultrasound and the absence of thyroid auto-antibodies ruled out other forms of hypothyroidism. We began therapy with oral propranolol at an initial dose of 1 mg/Kg with subsequent dose escalation to 2 mg/kg after five days. The treatment was well tolerated with no documented side effects. No thyroid hormone replacement therapy was required as after two weeks of therapy, TSH level normalized. After 1 month of therapy, ultrasound showed a marked reduction of the hepatic lesions. After 1 year, liver and skin hemangiomas were no longer observable and TSH remained normal, so we discontinued oral propranolol.

**Table 1:** Thyroid blood tests of our case at diagnosis, after 14 days and 30 days of propranolol therapy

	Normal value for age	At diagnosis (2 months of age)	After 14 days of therapy	After 30 days of therapy
**TSH (microU/ml)**	0.25-5	33.05	5	1.88
**fT4 (pg/ml)**	5.5–15	16.8	13.9	10.8
**fT3 (pg/ml)**	2.4-5	3.7	3.1	3.3
**rT3 (ng/ml)**	0.5–0.95	2	0.67	--

The demographic and clinical features of the cases identified in our literature review are shown in Table 2. 31 out of 64 (48.43%) of patients are female and the median age at diagnosis is 2 months. Death is reported in 7/64 patients (10.93%). The laboratory profile shows very high TSH levels at diagnosis, with a mean value of 145.5 mU/L. As regards the tumor-directed therapy, the use of different treatments is reported including corticosteroids, interferon, vincristine, cyclophosphamide, propranolol, surgery and liver transplantation. 38/64 patients (59.37%) received propranolol, either alone (20/38, 52.63%) or in combination with other treatments. 12/64 patients (18.75%) underwent surgical treatment or liver transplant.

Treatment of hypothyroidism consisted in the administration of LT4 and/or liothyronine. Among the 56 patients for whom we have data regarding the therapy, 52/56 (92.85%) received LT4. The mean maximum dose of LT4 alone was 24.04 mcg/kg/die; reported dosages ranged between 1 and 110 mcg/kg/d. The association of liothyronine was required in 15/52 (28.8%) patients. In 1 case, only liothyronine was used (Table 2, case n.45).

**Table 2:** Review of case reports and case series reporting children with consumptive hypothyroidism due to infantile hepatic hemangioma (2000–2023)

CASE *N*.	AUTHOR, YEAR	Sex	Age at diagnosis (months)	Outco me	TSH at diagnos is (microU/mL, *n*.v. 0,25 − 5)	fT4 at diagnosis (pg/ml, *n*.v. 5,5–15)	IHH Therapy	LT4 therapy	LT4 max dosage (mcg/kg/ die) *	Liothyron ine therapy
1	Huang, 2000 [11]	M	1.5	Dead	177	25	St–Int-Sur	Yes	7.6	Yes
2	Mason, 2001 [23]	M	1.75	Alive	203	4.6	St-Int-Sur	Yes	7	Yes
3	Ayling, 2001 [24]	F	2	Dead	103	13.2	Tr	Yes	°	No
4	Ayling, 2001 [24]	M	0.3	Alive	35	3.9	Tr	Yes	°	No
5	Ayling, 2001 [24]	M	1.25	Alive	200	20.1	Sur	Yes	°	No
6	Ayling, 2001 [24]	F	4	Alive	475	3.9	Sur	Yes	°	No
7	Ayling, 2001 [24]	F	4	Alive	16	9.3	Sur	°	°	°
8	Ayling, 2001 [24]	F	4	Alive	°	21.7	Sur	°	°	°
9	Ayling, 2001 [24]	M	5	Alive	40	°	Sur	°	°	°
10	Ayling, 2001 [24]	M	0.5	Dead	°	38	Sur	°	°	°
11	Konrad, 2003 [25]	M	2	Alive	100	3.9	Pr	Yes	28	No
12	Güven, 2005 [26]	F	0	Alive	100	°	St	Yes	19.8	Yes
13	Ho, 2005 [27]	F	2.5	Alive	90	16.7	St	Yes	°	Yes
14	Lee, 2006 [28]	F	1.5	Alive	182	0.8	St-Vin-Int- Cyc-Tr	Yes	15	No
15	Balazs, 2007 [29]	F	1.5	Alive	182	8	St–Int–Tr	Yes	75	Yes
16	Kalpatthi, 2007 [30]	M	4	Alive	53.3	13	St	Yes	°	No
17	Cho, 2008 [31]	M	7	Alive	18.98	16.3	St–Vin	Yes	5.4	No
18	Mouat, 2008 [32]	F	0.75	Alive	17	16.5	St	Yes	25	No
19	Çetinkaya, 2010 [33]	M	0	Alive	150	24.1	St – Int	Yes	22	Yes
20	Peters, 2010 [34]	M	1	Alive	66.2	13.1	St – Vin	Yes	20	Yes
21	Bessho, 2010 [35]	F	0	Alive	45.2	18	St – Int – Sur	Yes	7.5	No
22	Mazereeuw-Hautier, 2010 [15]	°	°	Alive	°	°	Pr	°	°	No
23	Mazereeuw-Hautier, 2010 [15]	°	°	Alive	°	°	Pr – St– Vin	°	°	No
24	Mazereeuw-Hautier, 2010 [15]	°	°	Alive	°	°	Pr – St	°	°	No
25	Marsciani, 2010 [16]	F	2	Alive	33.8	17.5	Pr – St– Vin - Cyc	Yes	1	No
26	Jassam, 2011 [36]	M	2	Dead	138	12.9	St – Int– Sur	Yes	10	No
27	Yeh, 2011 [37]	F	1.5	Alive	68	°	St – Vin	Yes	°	Yes
28	Yeh, 2011 [37]	M	1	Alive	19.8	24	St – Vin	No	-	No
29	Yeh, 2011 [37]	M	1	Alive	31.4	°	Pr – St	Yes	°	No
30	Yeh, 2011 [37]	F	0.5	Alive	55.8	°	Pr – St– Vin	Yes	°	Yes
31	Imteyaz, 2011 [38]	F	4	Alive	14.2	27	St	Yes	°	Yes
32	Mhanna, 2011 [39]	F	1.5	Alive	9.47	12.2	Pr	Yes	°	No
33	Mhanna, 2011 [39]	M	3	Alive	°	in range	Pr	Yes	°	No
34	Vergine, 2012 [17]	F	2	Alive	40	18.5	Pr – St– Vin	Yes	2	No
35	Avagyan, 2013 [40]	F	0.5	Alive	65.1	in range	Pr	Yes	30	No
36	Wijeratne, 2014 [41]	M	1.5	Alive	37.7	13.9	Pr – St	Yes	16	No
37	Sun, 2014 [42]	F	0.5	Dead	°	°	St	Yes	°	No
38	Wasserman, 2015 [43]	F	2	Alive	123	7.2	Pr – St– Vin	Yes	21	Yes
39	Emir, 2016 [44]	F	0.5	Dead	74.2	13	Pr – St	Yes	18.5	No
40	Özdemir, 2017 [45]	M	4	Alive	177	10.7	Pr – St	Yes	°	No
41	Takai, 2017 [46]	M	0.25	Alive	54.7	10.7	Pr – St	Yes	°	No
42	Weber Pasa, 2017 [47]	F	0	Alive	24	4.6	Vin	Yes	110	Yes
43	Al Tasseh, 2017 [48]	M	3.5	Alive	220	11.6	Pr	Yes	25	No
44	Nguyen, 2017 [49]	M	2	Alive	54	°	Pr	No	-	No
45	Higuchi, 2017 [50]	M	2	Alive	17.7	14.8	Pr	No	-	Yes
46	Campbell, 2018 [51]	F	0.5	Alive	115.4	5.5	Pr	Yes	9.6	No
47	Simsek, 2018 [52]	M	4	Alive	177	12.3	Pr – St	Yes	35	No
48	Igarashi, 2018 [53]	M	0	Alive	153	8.8	Pr	Yes	12.5	No
49	Al-Ghamdi, 2018 [54]	M	2	Alive	281	8	Pr	Yes	9.6	No
50	Acharya, 2019 [55]	F	0.5	Alive	100	9	Pr – St	Yes	25	No
51	Osada 2019 [56]	M	4	Alive	561	11	Pr	Yes	13	Yes
52	Yang, 2019 [57]	F	26	Dead	°	°	Pr - St– Vin	Yes	°	No
53	Yang, 2019 [57]	F	1.5	Alive	°	°	Pr	Yes	°	No
54	Yang, 2019 [57]	F	1.8	Alive	°	°	Pr	Yes	°	No
55	Kim, 2020 [58]	M	1	Alive	100	5.9	Pr – St	Yes	7	No
56	Joshi, 2020 [59]	F	3	Alive	75	11.3	Pr – St– Int	Yes	33	Yes
57	Verma, 2020 [60]	F	4	Alive	17.5	°	Pr	Yes	9.1	No
58	Zheng, 2021 [61]	F	2	Alive	150	9.4	Pr	Yes	20	No
59	Siano, 2022 [20]	F	3	Alive	58.7	°	Pr	Yes	10	No
60	Siano, 2022 [20]	M	1	Alive	36.7	°	Pr	Yes	12	No
61	Siano, 2022 [20]	M	6	Alive	60.7	°	Pr - Sur	Yes	15	No
62	Numazawa, 2022 [62]	M	1	Alive	63.9	13.2	Pr - St	Yes	7.5	Yes
63	Morais, 2023 [63]	F	3	Alive	27.1	13.1	Pr	Yes	°	No
64	Our case	M	1.5	Alive	33.5	16.8	Pr	No	-	No

Abbreviations: F, female; M, male; n.v., normal value; Pr, propranolol; St, steroid, Vin, vincristine; Inf, interferon; Cyt, Cytoxan; Cyc – Cyclophosphamide; Sur, surgical treatment; Tr, transplantation; °, missing data

* Dosage is expressed in microg/kg/d when the pro kilo dosage was published or when the daily dose and the patient weight were published

## Discussion and conclusions

IHH is a visceral neoplasm, often associated with cutaneous IH, which can lead to serious and fatal complications. Early diagnosis of IHH allows for rapid initiation of therapy and reduces IHH mortality. The American Academy of Pediatrics recommends screening with abdominal ultrasound any child with 5 or more cutaneous IH to rule out visceral involvement [2]. Abdominal ultrasound can also be considered in children with fewer than 5 cutaneous IH, especially if associated with systemic impairment [20], even though a cut-off of 5 cutaneous IH has recently been confirmed to be the most appropriate threshold for abdominal ultrasonography screening [21]. 

Virtually all diffuse IHH can cause CH, with variable severity depending on the extension of the neoplasm. [1]Thyroid abnormalities may not be present at the time of neonatal screening as they occur when the neoplasm grows in the first few weeks of life. For this reason, we recommend investigating thyroid function in all infants with IHH, given the critical role of thyroid hormones in the development of the central nervous system in the first years of life.

Our review shows an increasing use of propranolol in the treatment of IHH, which is in fact supported by literature data regarding the efficacy of propranolol in reducing IHH size and complications [10, 15–17]. Traditional medical treatments for IHH (systemic corticosteroids, chemotherapy, hepatic surgery or liver transplantation) are of inconsistent efficacy and cause significant side effects [1, 17]. As evidence of this, among the reviewed cases, most of the patients requiring surgery or liver transplantation were diagnosed before 2011 and were not treated with propranolol. Also, the number of patients who died due to IHH complications is higher among those not treated with propranolol (5 vs. 2).

LT4 replacement therapy in CH often consists of very high doses of LT4, and in severe cases of the use of liothyronine in combination has been proposed [12]. This is due to the peripheral conversion to inactive forms (rT3) by Deiodinase 3, overexpressed in the hemangioma endothelium. Indeed, the reviewed cases received a mean dose of LT4 of 24.04 mcg/kg/day (range 1-110 mcg/kg/day), higher than the dose of 10–15 mcg/Kg/day usually adopted for primary congenital hypothyroidism [22]. 

An important observation regarding the treatment of CH is that patients who received propranolol required a mean dose of LT4 of 18.31 mcg/kg/d to correct hypothyroidism, while patients who received other treatments required higher doses of LT4, with a mean dose of 35.5 mcg/d. This suggests that propranolol not only effectively treats IHH, but also helps restore the euthyroid state by reducing tumor size. Moreover, 4 patients out of 64 (6.25%), including our case, did not require any hormone replacement treatment. These patients were all aged less than or equal to 2 months at diagnosis. This suggests that early recognition of the IHH and prompt start of propranolol therapy can effectively treat subsequent CH without the need for hormone replacement therapy. Obviously, it is essential to follow the patient with a close endocrinological follow-up.

In the presence of 5 or more cutaneous hemangiomas, an abdominal ultrasound is recommended to rule out liver involvement. When the diagnosis of IHH is confirmed, it is important to test thyroid function to promptly diagnose CH. Early therapy with propranolol reduces hepatic lesions and the occurrence of possible complications. Furthermore, it may allow to avoid the need for hormone replacement therapy. In the case of CH, careful monitoring of thyroid function is essential, and hormonal replacement therapy should be started when necessary.

## Data Availability

All data generated or analyzed during this study are included in this article. Further enquiries can be directed to the corresponding author.

## Abbreviations

CH: Consumptive hypothyroidism
D3: Type 3 deiodinase
IH: Infantile hemangioma
IHH: Infantile hepatic hemangioma
